# 

**Published:** 1878-03

**Authors:** 


### Contents of March Number.

### ORIGINAL.

ART. I.—Clinical Lecture on Club-Foot. By Prof. J. F. Miner, M. D. 281

### CORRESPONDENCE.

Letter from Berlin. C. F. A. Nichell, M. D.,........................ 290
Dilatation of Urethra by the Urine. H. B. Osborne, M. D............. 293
Fracture Lower Jaw in an Infant. Geo. A. Mursick, M. D.,... ....... 295
Statement from Prof. A. F. A. King, M. D.,........................ 296

### MISCELLANEOUS.

Aphorisms in Fracture. By R. O. Cowling, M. D.,.................. 297
Address of Dr. Frank H. Hamilton,........ ...................... 306
Dialysed Iron in Arsenical Poisoning.............................. 313

### EDITORIAL.

Thirty-Second Annual Commencement of Medical Department of University of Buffalo. Meeting of the Alumni, ..................... 313

OBITUARY.—Prof. Milton G. Potter, M. D.,......................... 316
Prof. E. R. Peaslee, M. D.,............................ 317

BOOKS REVIEWED:

Modern Medical Therapeutics. Modern Surgical Therapeutics. By Geo. H. Napheys, A. M., M. D.,................. 319
Public Hygiene in America. By Henry J. Bowditch, M. D., 319

Books and Pamphlets Received,.................................... 320

